# Dynamic Simulation and Parameter Analysis of Harpoon Capturing Space Debris

**DOI:** 10.3390/ma15248859

**Published:** 2022-12-12

**Authors:** Chunbo Wu, Shuai Yue, Wenhui Shi, Mengsheng Li, Zhonghua Du, Zhi Liu

**Affiliations:** 1 School of Mechanical Engineering, Nanjing University of Science and Technology, Nanjing 210094, China; 2 Shanghai Key Laboratory of Spacecraft Mechanism, Shanghai 201108, China; 3 Aerospace System Engineering Shanghai, Shanghai 201109, China

**Keywords:** space debris, harpoon, dynamic analysis, numerical simulation

## Abstract

This paper aims to study the penetration effect of harpoons on space debris to ensure the sustainable development of the space environment and solve the increasingly serious space debris problem. Firstly, a harpoon system was designed to capture space debris. Secondly, based on the Johnson–Cook dynamic constitutive model and fracture failure criterion, the finite element models of aluminum alloy plates were established. Then, the ballistic limit theory for the aluminum alloy target predicted the minimum launch velocity of the harpoon. Finally, the validation experiment was set up to verify the correctness of the model. The results show that the error between the simulation results of the speed for the harpoon embedded in the target and the theoretical results of the ballistic limit is 9.1%, which provides guidance for active space debris removal technology.

## 1. Introduction

The increasing number of space debris has seriously affected human space activities [1]. According to data from the U.S. Space Surveillance Network (SSN), with long-term monitoring of targets above 10 cm in low-Earth orbit and targets above 1 m in geostationary orbit, it is known that the number of objects in Earth’s orbit has surged over the last two decades, from approximately 11,000 in 2000 to nearly 20,000 in 2020 [2,3]. At present, many countries are actively exploring feasible solutions and providing appropriate policy and financial support for research [4]. As for the space debris removal system’s scheme selection, all countries unanimously prefer the active debris removal scheme. The working process was to rely on the tracking and positioning system to gradually approach the target debris without altitude control ability for capture; then rely on the orbital transfer system to off-orbit the target [5]. The existing capture technologies mainly include the mechanical arm, bionic capture, space harpoon and magnetic gravity capture [6]. The researchers proposed a multi-arm manipulator, such as the SUMO Universal Orbit Modification Spacecraft Project, supported by the U.S. Department of Defense [7], which is equipped with three 7-DOF manipulators. At the end of each manipulator, the tool could be switched through replaceable modules in orbit, and the objects of capture were the satellite arrow butt ring and separation bolt of the target star. However, this method was too expensive and had too few types of capture. Lin et al. [8] established the micro-contact principle between the elastic sphere and the plane and used it to describe the dynamic characteristics of the adhesion desorption process between the gecko, such as the polyurethane setae and contact surface. This principle was used to capture space objects. This capture method requires the surface roughness of the target object. The on-orbit ELSA-d conducted manual or semi-autonomous rendezvous of cooperative targets and captured the different poses of the targets. The ELSA-d mission demonstrated future space-efficient operations techniques for space cooperation goals [9]. This capture method was mainly aimed at space cooperation objectives. Campbell et al. [10] and Aglietti et al. [11] analyzed harpoons’ velocity during harpoon impact experiments on aluminum honeycomb panels in the Remove DEBRIS project. Mataki et al. [12] studied the influence of harpoon tip shape, launch velocity, incidence angle, low-temperature environment and self-locking structure on penetration results, but they lacked a study of ovoid harpoons. Wang et al. [13], Fras et al. [14], Kpenyigba et al. [15], and Deng et al. [16] studied the impact of blunt, hemispherical and bow-shaped projectiles at high intensities. The ballistic terminal velocity and target damage form of the three structures of armored steel, 2024-T351 aluminum alloy plate, low carbon steel plate and 6061-T651 aluminum alloy sheet, are made by simulation and experiment. They studied low-velocity intrusion into aluminum alloy target plates less.

The current study focuses on the harpoon penetration of aluminum honeycomb panels, high-speed projectile penetration of aluminum target panels and the damage effects of different shapes of harpoons. Little research has been conducted on the low-velocity penetration of ovoid harpoons into aluminum alloy target plates.

Following these investigations, the model of an oval harpoon penetrating target is proposed. The primary purpose is to evaluate the penetration effect and explain the behavior of aluminum alloy materials under the impact condition of the harpoon. The dynamic model of the harpoons’ impact on the target plate is established and coupled with the Johnson–Cook material model. The impact test of the harpoon penetrating the target is carried out to support this research. The simulation and experimental results at various launch speeds are compared to verify the correctness of the simulation model.

The structure of the paper is as follows: Section 1 outlines the relevant research and the main contributions of this paper; Section 2 studies the oval harpoon’s design, the target plate’s material model and the ballistic limit theory; Section 3 introduces the simulation model of the harpoon’s target, the simulation analysis of various velocity parameters and the experiments of the harpoon penetrating the target at various velocities.

## 2. Materials and Methods

This section mainly introduces the detailed structural design of the harpoon, the material constitutive model of the harpoon and the target plate and the establishment of an analytical model for predicting the ballistic limit velocity.

### 2.1. Harpoon and Target Plate Models

**Figure 1:**
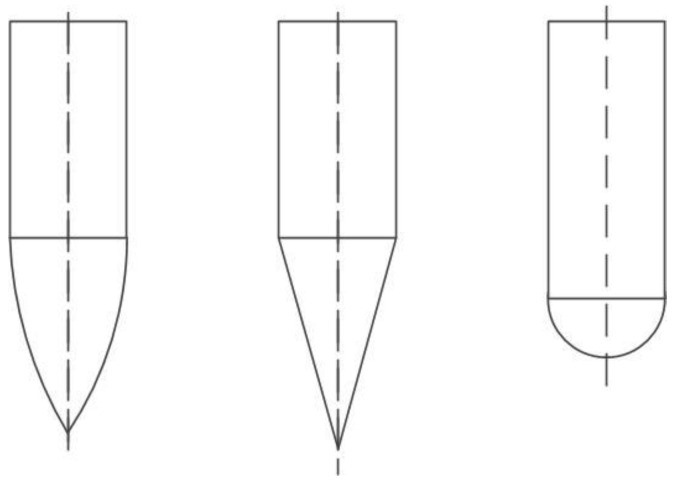
Different harpoon head shapes.

Whether the structural parameter design and material selection of the harpoon is correct has a great impact on the penetration performance of the harpoon. The shape of the harpoon’s head varies in penetration structure, complicating the research. Standard harpoons have conical, oval and semicircular heads, as shown in Figure 1.

**Figure 2:**
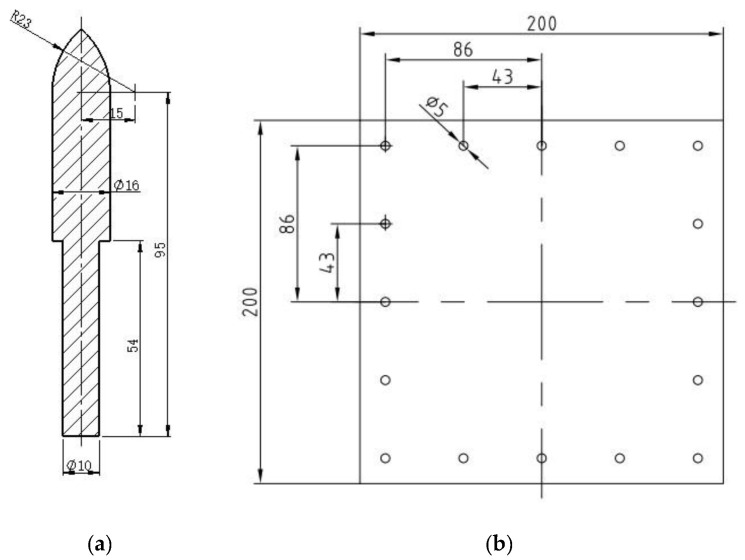
Structural model (the unit of measurement is mm). (**a**) Harpoon and (**b**) target board.

The penetration capability of three different harpoon head shapes (oval head, tapered head and hemispherical head) was compared, and the oval harpoon head with the best penetration performance was obtained [17]. The residual velocity of the ovoid harpoon is the highest when the overall harpoon velocity and mass are kept unchanged. Therefore, the shape of the head of the harpoon was chosen to be oval, as shown in Figure 2a.

The central part of the harpoon ensures the penetration ability and emission strength, so the alloy steel of 30CrMnSiA was selected. 30CrMnSiA is a medium carbon alloy steel with good processability. After quenching and tempering, the material had high strength and sufficient toughness to meet the emission strength requirements. The material of other parts was mainly aluminum alloy, which can reduce the overall weight.

2A12 aluminum alloy is often used as the skin of aerospace materials and other structures. Therefore, the target of the harpoon’s penetration was a 2A12 aluminum alloy target plate with a thickness of 2 mm, as shown in Figure 2b. The size of the target plate was 200 × 200 mm, and it was fixed on the bracket by M5 bolts.

### 2.2. Target Material Model

The impact dynamics are numerically calculated in ABAQUS 2016. The Johnson–Cook dynamic constitutive model and fracture failure criterion are the most used dynamic constitutive and dynamic failure criteria for metals.

When the harpoon penetrates the target plate, the material undergoes large deformation and a high strain rate, and the temperature of the material also sharply increases. The model utilizes the von Mises yield surface and flow law and considers factors of the material, such as material strain, strain rate hardening and temperature softening, which are assumed to be decoupled. The expression is:

(1) $$\bar{\sigma }=f\left({\bar{\varepsilon }}^{p},{\dot{\bar{\varepsilon }}}^{p},T\right)=f_{1}\left({\bar{\varepsilon }}^{p}\right)f_{1}\left({\dot{\bar{\varepsilon }}}^{p}\right)f_{1}\left(T\right)$$

In the model, the strain hardening and temperature softening factors are in the form of a power function, and the strain rate hardening factor is in the logarithmic form. The formula is:

(2) $$\bar{\sigma }=\left[A+B{\left({\bar{\varepsilon }}^{p}\right)}^{n}\right]\underset{strain\;rate\;effect}{\underset{︸}{\left[1+C\ln\left(\frac{{\dot{\bar{\varepsilon }}}^{p}}{{\dot{\bar{\varepsilon }}}_{0}}\right)\right]}}\underset{temperature\;effect}{\underset{︸}{\left[1-{\left(\frac{T-T_{room}}{T_{melt}-T_{room}}\right)}^{m}\right]}}$$

where A is the initial dynamic yield strength, B is the strain rate hardening coefficient, n is the strain rate hardening index, C is the strain rate sensitivity coefficient, *m* is the softening index of temperature rise, $T_{melt}$ is the melting point temperature of the material, $T_{room}$ is the ambient temperature, $\bar{\sigma }$ is the von Mises yield stress, ${\bar{\varepsilon }}^{p}$ is the equivalent plastic strain, ${\dot{\bar{\varepsilon }}}^{p}$ is the plastic strain rate and ${\dot{\bar{\varepsilon }}}_{0}=1.0s^{-1}$ is the reference strain rate.

The Johnson–Cook failure criterion gives the relationship between material strain rate, stress triaxiality, temperature and material failure strain rate, and its expression is:

(3) $${\bar{\varepsilon }}^{f}=\left[D_{1}+D_{2}\exp\left(D_{3}\frac{p}{\sigma }\right)\right]\underset{strain\;rate\;effect}{\underset{︸}{\left[1+D_{4}\ln\left(\frac{{\dot{\varepsilon }}^{p}}{{\dot{\varepsilon }}_{0}}\right)\right]}}\underset{temperature\;effect}{\underset{︸}{\left[1+D_{5}{\left(\frac{T-T_{room}}{T_{melt}-T_{room}}\right)}^{m}\right]}}$$

where ${\bar{\varepsilon }}^{f}$ is equivalent failure strain, $D_{1},D_{2},D_{3}$ is the stress triaxiality-related parameter, $D_{4}$ is the strain rate influence coefficient and $D_{5}$ is the temperature influence coefficient.

### 2.3. Ballistic Limit Theory

In order to facilitate the harpoon’s penetration or embedding of the target, it is necessary to determine the launch speed of the harpoon in advance. The penetration behavior of the harpoon into the target plate is very complex, and its acceleration is constantly changing, so it is challenging to establish an analytical model of the harpoon penetrating the target plate’s finite thickness. By studying the ballistic limits of aluminum alloy targets, it is possible to predict the rate of launch required for the harpoon to penetrate the target. The ballistic limit theory is mainly based on the energy law and the law of conservation of momentum to establish its theoretical models. The Recht and Ipson (RI for short) is the simplest and most widely used model [18].

The RI model assumes that the harpoon’s required kinetic energy to penetrate the target plate is equal to the harpoon’s required energy to form a channel. Therefore, the kinetic energy of the harpoon before and after penetrating the target plate has the following relationship with the harpoon’s effect on the target plate:

(4) $$\frac{1}{2}MV_{0}^{2}=\frac{1}{2}MV_{r}^{2}+W_{P}$$

where $V_{0}$ and $V_{r}$ are the impact and residual velocities of the projectile, respectively. The ballistic limit speed, $V_{b}$, is the impact speed when the residual speed is zero, so there is the following equation:

(5) $$\frac{1}{2}MV_{b}^{2}=W_{P}$$

By combining these two equations, it can be concluded that:

(6) $$V_{r}^{2}=V_{0}^{2}-V_{b}^{2}$$

Its dimensionless form is:

(7) $$\frac{V_{r}}{V_{b}}=\sqrt{{\left(\frac{V_{0}}{V_{b}}\right)}^{2}-1}$$

It is necessary to ensure that the flying spear is inserted or has penetrated the target to prevent the threat of the flying spear rebounding to the launching platform. Hence, the launching speed of the flying spear needs to be slightly higher than the optimal speed. According to Rosenberg’s research [19], the calculation results of the RI model are very consistent with the test data, and this equation can explain the relationship between the impact and the residual velocities of the spear–target combination. The RI model considers that the energy required to penetrate a given target is almost independent of the impact velocity, and it gradually approaches with the increase of the spear’s impact velocity. Although the model is concise, it fails to combine the physical properties of the materials. Rosenberg established an analysis model to predict the final ballistic velocity by studying the equivalent resistance exerted by the flying spear during penetration [20]. The basic idea is to use equivalent resistance stress to express the resistance varying with time in actual penetration. That is, the kinetic energy loss of the spear caused by constant and actual stress is the same [21]. The motion equation of a harpoon with equivalent stress, $\sigma_{r}$, penetrating the target plate, H, can be defined as:

(8) $$F=M\frac{dV}{dt}=MV\frac{dV}{dx}=\pi r^{2}\sigma_{r}$$

where M and r are the mass and radius of the projectile, which are obtained by integrating the boundary conditions from x=0, $V_{0}=V_{b}$ to x=H and V=0.

(9) $$\frac{MV_{b}^{2}}{2}=\pi r^{2}H\sigma_{r}$$

The ballistic limit speed is:

(10) $$V_{b}=\sqrt{\frac{2\pi r^{2}H\sigma_{r}}{M}}$$

It can be seen from the above formula that if the $\sigma_{r}$ value is known, the ballistic limit velocity of any harpoon–target combination can be obtained. In principle, the $V_{b}$ value can be obtained without testing, and the $\sigma_{r}$ value is closely related to the target plate material strength limit, $Y_{t}$, and target plate thickness, HH. Rosenberg determines the dependence, $\sigma_{r}$, on the target plate’s strength and thickness on this basis and establishes the relationship between the relative equivalent stress $\left(\sigma_{r}/Y_{t}\right)$ and the relative thickness H/D (where D represents the harpoon’s diameter). For the thin targets using (H/D≤1/3), the relationship between $\left(\sigma_{r}/Y_{t}\right)$ and H/D is proportional:

(11) $$\frac{\sigma_{r}}{Y_{t}}=\frac{2}{3}+4\left(H/D\right)$$

The diameter of the harpoon used to calculate the embedded ballistic limit is $D_{1}=16\;\mathrm{mm}$. The harpoon’s mass is M=120.0 g. The target plate material is 2A12 aluminum alloy; its strength limit is $Y_{t}=400$ MPa, and its thickness is 2 mm. 2A12 aluminum alloy is commonly used as an aerospace material, such as a satellite protective plate. It is calculated that the embedding speed of a single-rod flying spear is $V_{b}=55.93$ m/s. Therefore, the best launch speed of the harpoon is at least $V_{0}=55.93$ m/s.

## 3. Results and Discussion

This section is mainly based on ABAQUS/Explicit to establish the simulation model of the harpoon penetrating the aluminum alloy target. The simulation model is validated by designing the ground test of the harpoon penetrating the target.

### 3.1. Simulation Model of the Harpoon Penetrating the Target

**Figure 3:**
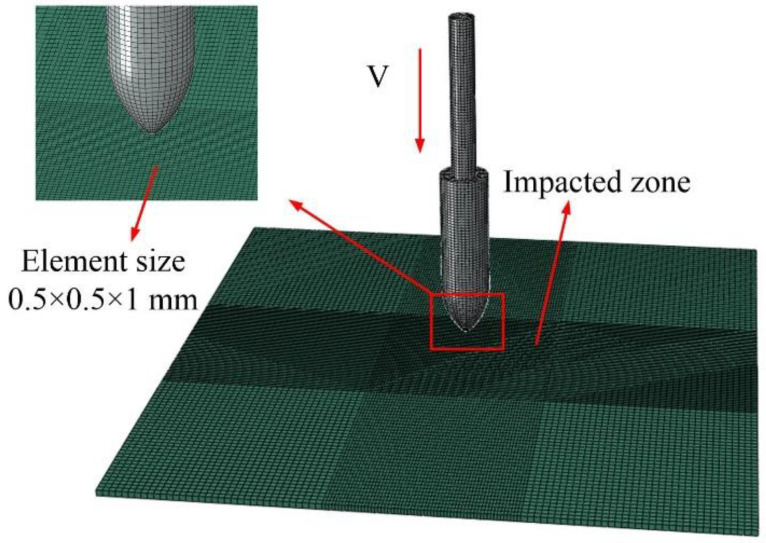
Harpoon penetration simulation.

In this section, the numerical simulation of the harpoon system is carried out. A simulation model of the harpoon penetrating plates was established in ABAQUS. The visualization of the model is shown in Figure 3. According to our observations, the harpoon did not experience visible deformation during the experiment, so, in the numerical model, they were modeled as rigid bodies and their element type was set as C3D8R. The layout of the target plates was the same as in the test conditions, and the area of the target plate was 200 × 200 mm. The target plate is made of 2A12 aluminum alloy 2 mm thick. It is necessary to refine the mesh in the central area of the target plate to increase the computational accuracy. The target plate is modeled as a solid unit, and the unit type is C3D8R.

In this paper, the material model of 2A12 aluminum alloy used the Johnson–Cook constitutive model. The material parameters are summarized in Table 1. In the table, ρ is the density, E is Young’s modulus and μ is Poisson’s ratio, which are the primary mechanical parameters of the material. Additionally, A is the dynamic yield strength, B is the strain rate hardening coefficient, n is the strain rate hardening index, C is the strain rate hardening coefficient and *m* is the temperature rise softening index, which are the parameters of the Johnson–Cook constitutive model. Finally, $D_{1}-D_{5}$ are the Johnson–Cook failure criterion parameters [22].

### 3.2. Multi-Velocity Simulation of the Harpoon Penetrating the Target

According to the above theory, the finite element model of the harpoon impacting the target plate was established.

**Figure 4:**
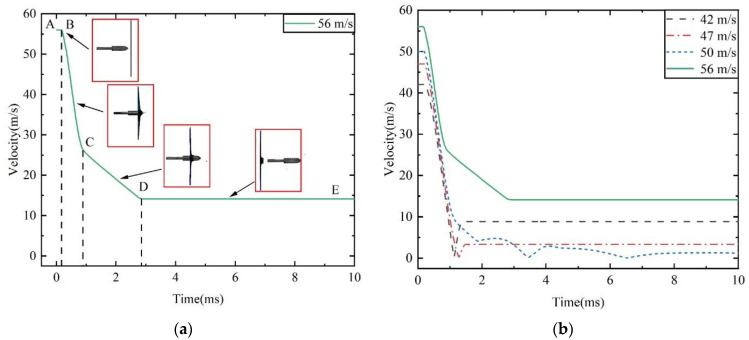
Velocity evolution curve. (**a**) Velocity evolution at 56 m/s of the initial velocity, and (**b**) velocity evolution at 42, 47, 50 and 56 m/s of initial velocities.

The impact results for the harpoon with an initial velocity of 56 m/s on the aluminum alloy target plate are shown in Figure 4a. The harpoon contacts the target plate at point B. When the harpoon head penetrates the target plate in the BC stage, its velocity decreases rapidly. The middle part of the harpoon passes through the target plate in the CD stage, and the velocity attenuation is mainly due to friction resistance. The harpoon leaves the target plate at point D, after which the remaining speed does not change.

Figure 4b shows the simulation results of other speeds. The harpoons with initial speeds of 42 and 47 m/s did not penetrate the target plate. After the harpoon hits the target plate, its speed rapidly decreases to 0, and then it is bounced off by the target plate. When the launching speed of the harpoon is 50 m/s, it hits the target plate and remains in the middle, and the speed oscillates back and forth.

**Figure 5:**
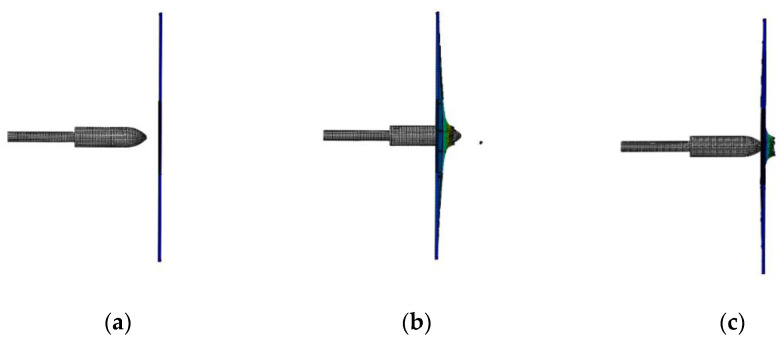
Simulation results for a launch speed of 42 m/s: (**a**) before penetration; (**b**) penetrating; (**c**) no penetration.

**Figure 6:**
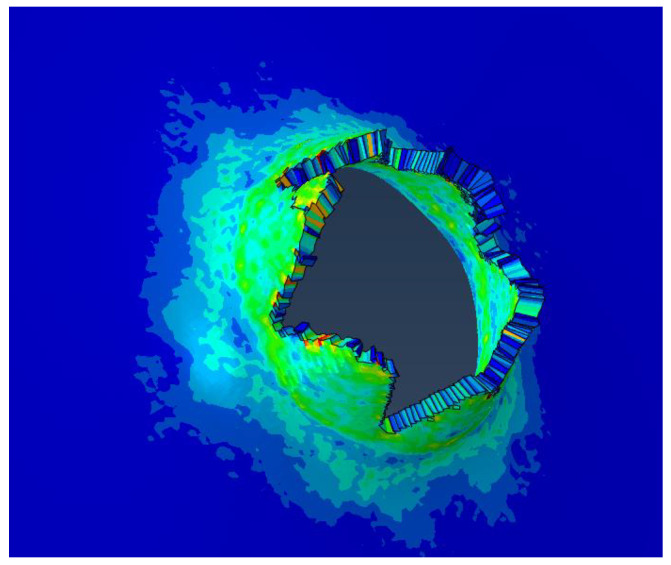
Details of target plate damage for a launch speed of 42 m/s.

When the launching speed of the harpoon is 42 m/s, the harpoon fails to penetrate the target plate, as shown in Figure 5. When the harpoon penetrates the target, it first causes the middle surface of the target to deform and bulge. When moving forward, the tip of the harpoon pierces the target plate, and the target plate forms petal cracking at the hole, as shown in Figure 6. The diameter of the simulated hole is 18.1 mm.

**Figure 7:**
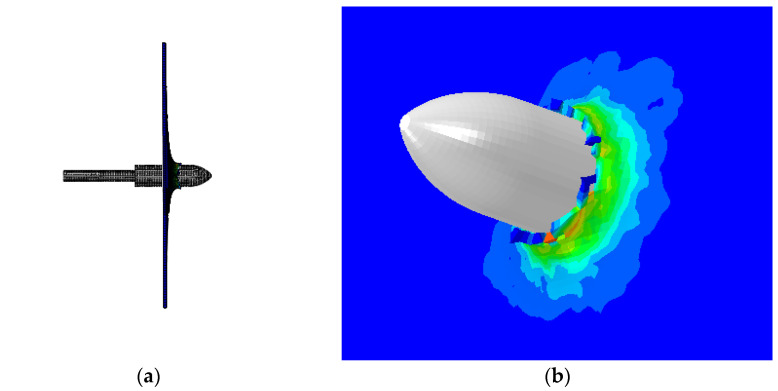
Simulation results of 50 m/s initial velocity: (**a**) simulation results side view, and (**b**) embed details.

When the projection speed of the harpoon is 50 m/s, it is embedded in the target plate, as shown in Figure 7a. The connection details of the harpoon and target plate are shown in Figure 7b. The error between the simulation results of the harpoon’s speed embedding into the target and the theoretical results of the ballistic limit is 9.1%.

**Figure 8:**
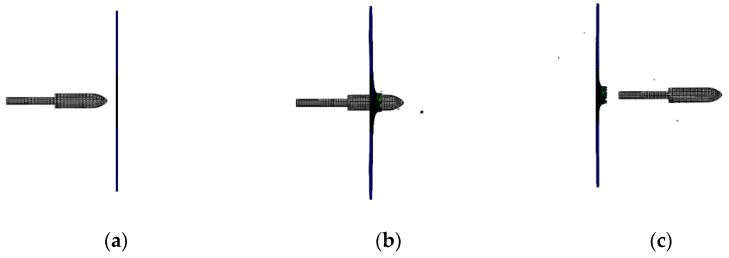
Simulation results for a launch speed of 56 m/s: (**a**) before penetration; (**b**) penetrating; (**c**) after penetration.

**Figure 9:**
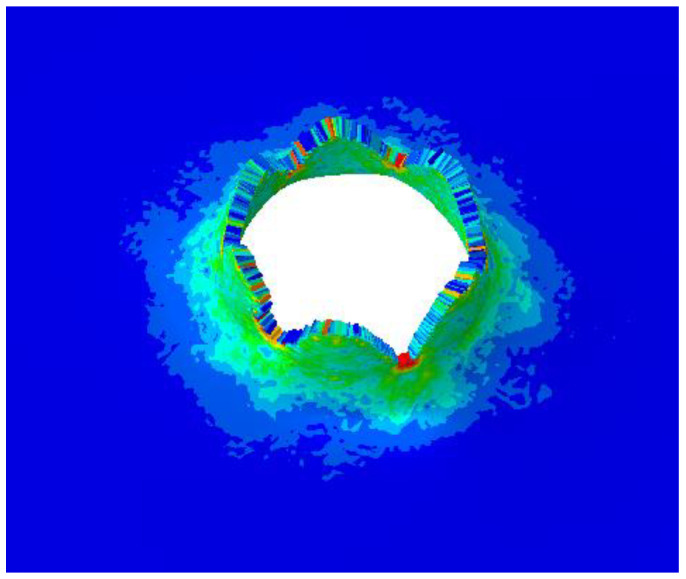
Details of target plate damage from a launch speed of 56 m/s.

When the launching speed of the harpoon is 56 m/s, the harpoon penetrates the target plate, as shown in Figure 8. The harpoon pierces the target plate, which forms petal-shaped cracks at the hole, as shown in Figure 9. The diameter of the simulated hole is 20.1 mm.

### 3.3. Multi-Velocity Experiment of the Harpoon Penetrating the Target

**Figure 10:**
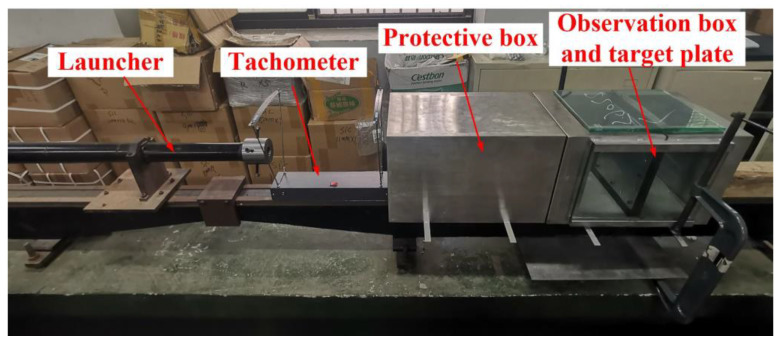
Harpoon impact test layout.

In order to verify the simulation model, the experimental design was carried out for the harpoon impacting the target plate. The layout of the test site is shown in Figure 10. The test layout includes a launcher, tachometer, protection box, observation box and target plate from left to right. The harpoon is placed inside the launcher. The transmitter is connected to the high-pressure gas cylinder, and the controller controls the gas emission pressure and emission switch. The whole test process was recorded by a high-speed camera. The shape and quality of the harpoon used in this test are the same as the simulation model. The aluminum plate struck by the harpoon is made of 2A12 aluminum alloy with a thickness of 2 mm.

In this paper, four experiments of a harpoon penetrating aluminum plates at different velocities are carried out. The dynamic behavior of the harpoon penetrating the target is recorded by a high-speed camera. The experimental results are shown in Table 2.

**Figure 11:**
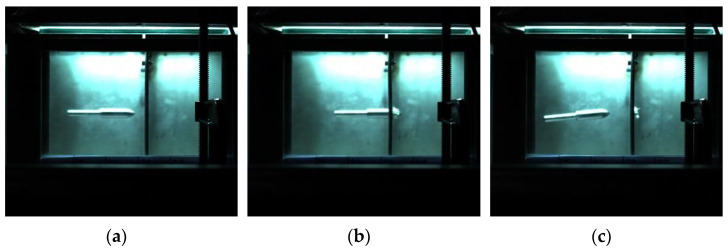
Experimental results for the launch speed of 42 m/s: (**a**) before penetration; (**b**) penetrating; (**c**) after collision.

The impact process of the harpoon with a launching speed of 42 m/s is shown in Figure 11. The flying attitude of the harpoon is stable before it hits the target plate. The harpoon cannot penetrate the target plate due to its low speed.

**Figure 12:**
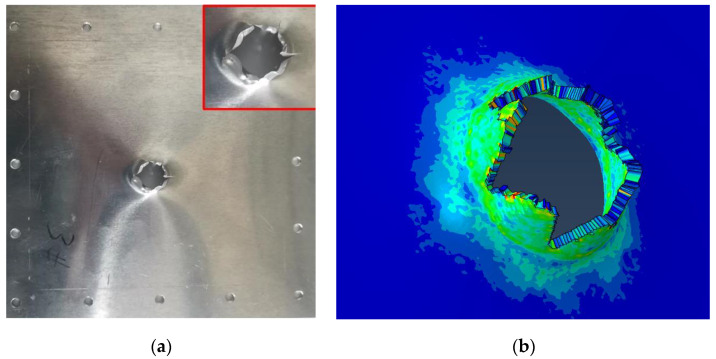
Simulation and experiment comparison: (**a**) experiment results; (**b**) simulation results.

The harpoon rebounds after the impact tear create a small hole. The failure of the target plate in the shape of petal cracking is a typical thin plate failure form, as shown in Figure 12. The diameter of the target plate in the test is 16.4 mm. When the speed of the harpoon is 42 m/s, the error of hole breaking is 9.34% between the simulation and test results.

**Figure 13:**
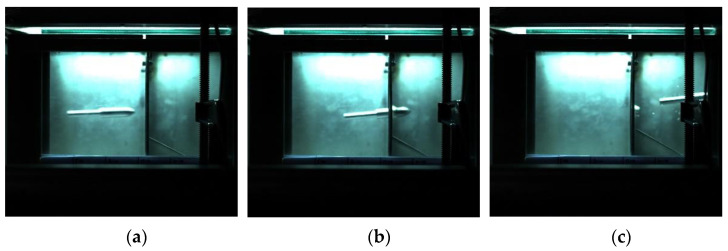
Experimental results for the launch speed of 56 m/s: (**a**) before penetration; (**b**) penetrating; (**c**) after penetration.

The impact process of the harpoon with a launching speed of 56 m/s is shown in Figure 13. At this speed, both the experimental and simulated harpoons penetrated the target smoothly.

**Figure 14:**
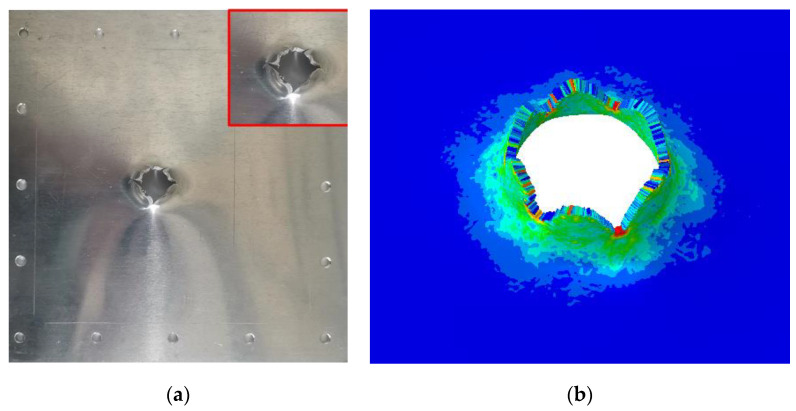
Simulation and experiment comparison: (**a**) experiment results; (**b**) simulation results.

The harpoon penetrates the target plate after impact, and the target plate forms a petal-shaped opening, as shown in Figure 14. The diameter of the target plate in the test is 19.9 mm. When the speed of the harpoon is 56 m/s, the error of hole breaking is 1.0% between the simulation and test results.

The experimental results are consistent with the simulation results, indicating that the simulation model is correct. Through simulation, it can be found that the harpoon is embedded into the aluminum alloy target plate at a speed of 50 m/s. To ensure that the harpoon is accurately connected with the target, the launching speed of the harpoon should be at least 50 m/s.

## 4. Conclusions

In this paper, the laws of the harpoon penetrating the aluminum alloy target at low speeds are studied. A nonlinear transient dynamics model of the harpoon system is established based on the ABAQUS/Explicit method. A simulation analysis of the intrusion of the model at different velocities is carried out. The test results are also compared to determine the correctness of the simulation model and the effectiveness of the harpooning scheme. The main conclusions are as follows:

1. The ballistic limit theory was developed based on the energy law and the law of conservation of momentum and was used to predict the velocity limit of an elliptical harpoon penetrating a target. The error between the simulation results for the speed of the harpoon embedding into the target and the theoretical results of the ballistic limit is 9.1%
2. To verify the correctness of the simulation model of the harpoon’s penetration into the aluminum target plate, we designed a ground test of the harpoon penetrating the target plate with different velocities. The error of the hole diameter for the target plate with the launch speeds of 42 and 56 m/s is 9.34 and 1.0%, respectively.
3. Many structures in space debris are aluminum alloy plates. To ensure that the harpoon successfully catches 2A12 aluminum alloy that is 2 mm thick, the launch speed of the harpoon is at least 50 m/s.

## Figures and Tables

**Table 1:** Material parameters of the 2A12 aluminum alloy.

*ρ*/(kg·m^−3^)	*E*/GPa	*μ*	*A*/MPa	*B*/MPa	*n*
2770	71.7	0.33	400	424	0.350
*m*	*D*_1_	*D*_2_	*D*_3_	*D*_4_	*D*_5_
1.426	0.116	0.211	−2.172	0.012	−0.01256

**Table 2:** Experimental results.

Test No.	Target Plate Thickness (mm)	Weight (g)	Launch Velocity (m/s)	Experimental Result
F-01	2	111.9	28.6	No penetration
F-02	2	110.5	35.3	No penetration
F-03	2	112.5	42.0	No penetration
F-04	2	111.2	56.0	Penetration

## Data Availability

Not applicable.
